# Study on Virtual Experience Marketing Model Based on Augmented Reality: Museum Marketing (Example)

**DOI:** 10.1155/2022/2485460

**Published:** 2022-05-19

**Authors:** Ye Zhu, Chong Wang

**Affiliations:** ^1^School of Art and Design, Guilin University of Electronic Technology, Guilin 450305, China; ^2^School of Business, Guilin University of Electronic Technology, Guilin 450305, China

## Abstract

With the development of emerging digital technologies such as Augmented Reality and Artificial Intelligence, Augmented Reality (AR) technology-enabled experience marketing model can bring brand new virtual experience to the users, improve the brand attitudes of users, and increase the use and purchase intention of users. Based on the theoretical basis of experience marketing and AR, the AR application of Guilin Museum was designed and developed by using Unity as the software development tool and using AR Foundation as the AR development framework. The implementation of this application was mainly based on face detection and tracking, image detection, and tracking in the underlying API of AR Foundation. Subsequently, an AR virtual experience marketing model was constructed based on the Schmitt strategic experience module, and the usage data of AR applications were collected. Furthermore, the collected data were analyzed and evaluated using SPSS and AMOS software, and the relationships and influences of sensory experience, emotional experience, thinking experience, action experience, and association experience on the brand attitudes of users and use intention and purchase intention in AR application were tested.

## 1. Introduction

Experiential marketing is a new thinking and a new marketing mode in the era of experience economy [1]. Users can participate in the marketing process independently by creating sensory stimulation for users, real-time interaction led by emotional resonance [2]. Experience marketing provides diversified information for the public to make purchase decisions and transfers the experience value generated in the marketing processes to the intangible brand value [3]. The traditional marketing method considers public as the embodiment of “Rationality,” product-oriented, and focuses on displaying its functions and characteristics; experience marketing considers public as the embodiment of the combination of “Rationality” and “Sensibility,” users-oriented, fits with their senses and hearts, and focuses on creating unique consumption experience for users [4, 5]; impulsive purchase behaviors will be more possible if the experience degree and perceived value of users are higher [6].

With the development of digital technology, its innovative and interactive characteristics have created an ideal space for experiential marketing with a wider range at a deeper level. Particularly, the emerging Augmented Reality (AR) has expanded endless virtual interaction space for brands and users, of which the virtual experience marketing model has become a new “bridge” connecting the brand and public [7]. AR technology integrates digital information, virtual space, and the real world. AR application can make virtual content around the culture or products of Brand IP (Intellectual Property), provide the virtual experience to the users, and extend the single offline scene of experience marketing to the global perspective of online and offline linkage. AR experience marketing “leads” the consumption experience and creates virtual elements and content to attract users, achieve precision marketing and interactive marketing, touch the deep perception of users, and obtain high loyalty and satisfaction.

Particularly, the marketing concept can be used by profit-making organizations and nonprofit organizations, such as museums and churches. Museum is an important educational place used for providing services to public, and AR virtual experience marketing can be used as a new paradigm to promote museum culture with its characteristics of multiperception, immersion, and strong interaction. In order to promote the Guilin Museum and market cultural relic cards of the museum, this paper uses Unity as the software development tool and AR Foundation as the AR development framework to design and develop the museum AR application. ARKit, as the underlying API of AR Foundation, provides algorithms for face detection and tracking, image detection and tracking, and Blendshapes facial expression factor-driven model technology. Then, based on the Schmitt strategic experience module, an AR virtual experience marketing model was constructed, and an empirical analysis of the museum AR software was carried out to test the promoting effect of the museum AR application and the marketing effect of the cultural relic card.

## 2. Literature Review

### 2.1. Overview of Augmented Reality

**Figure 1:**
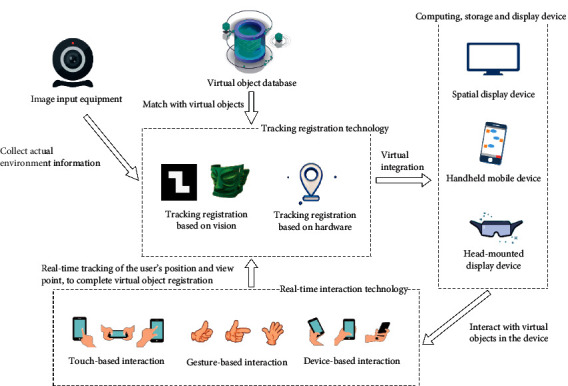
System architecture of Augmented Reality (the image materials in the figure are from the network.).

AR technology integrates virtual space and the real world to complete the effective interaction between reality and virtual space. The information of virtual space can feed back and strengthen the objects in real space and deepen the understanding of users about real space. Milgram and Kishino [8] proposed an AR theory, namely, “Reality-Virtual Continuum.” They believe that AR is between reality and virtual. Azuma's study shows that AR shall include three key parts: virtual-real fusion, real-time interaction, and registration tracking [9]. The AR system realizes the registration of virtual objects through real-time tracking of the surrounding environment, presents the scene of virtual-real fusion through display equipment, and realizes the interaction with virtual objects through real-time interaction technology. The overall architecture is shown in Figure 1.

#### 2.1.1. Tracking Registration Technology

Tracking registration, the core technology of AR system, refers to the technology that can realize the mapping of virtual and real space coordinate system by tracking the viewpoint and field of view of a user and superimpose virtual information in the real scenario according to the accurate spatial perspective relationship [10]. As for virtual objects rendered in real time in the AR system, all-round tracking and positioning with the real environment should be realized; since the user's vision is extremely sensitive to any positioning error, the greater visual error may affect the consistency and coordination of virtual-real combination and even affect the perception of the surrounding environment; therefore, AR has high requirements for registration precision. At present, there are three tracking registration technologies: vision-based tracking registration technology, hardware sensor-based tracking registration technology, and hybrid tracking registration technology [11].

#### 2.1.2. Real-Time Interaction Technology

AR real-time interaction mainly involves the interaction with 2D screen and control of 3D virtual objects. The scholars mainly focused on the control of 3D objects, such as the control of 6DOF (degree of freedom), including translation along the *XYZ* axis and pitching, yawing, and rolling rotation around the *XYZ* axis. AR system mainly involves the following interactions: touch-based interaction, air gesture-based interaction, and device-based interaction [12].

#### 2.1.3. Virtual-Real Fusion Technology

Virtual-real fusion refers to the display technology that seamlessly integrates virtual scenes with real ones. Augmented Reality system should ensure the consistency of virtual and real scenes, including geometric consistency, illumination consistency, and time consistency [13]. There are two types of fusion methods: optical perspective and video perspective. The video perspective method collects data from the real environment by camera, and the built-in information processing unit could recognize and processes the image and perform tracking registration of real objects with a variety of embedded sensors (such as the accelerometer, gyroscope, infrared sensor, and magnetometer) to integrate virtual images and real ones. The optical fusing device has the characteristics of partial transmittance and partial reflection, based on which, the user can directly observe the real world; at the same time, the virtual image generated by the system through calculation can be projected into the fusion device and reflected into the user's eyes; finally, the users may view the images of virtual-real fusion [14].

### 2.2. AR Foundation and ARKit Principle

Unity is committed to building a unified and open AR development platform which has a high support rate. By comparing the well-known AR software development kits (Software Development Kit, SDK) at home and abroad, this paper chooses AR Foundation of Unity as the development tool from the perspective of function realization and efficiency of development and chooses the IOS system as the publishing platform. Since AR Foundation only integrates a third-party AR SDK in Unity, the underlying implementation of AR applications developed based on the IOS system comes from the ARKit framework.

The ARKit framework of Apple Inc. based on the IOS system has many significant advantages and functional features. The main functions of ARKit include 2D image recognition and tracking, 3D object recognition and tracking, environmental probe, character occlusion, motion capture, face detection and tracking, and multiperson sharing. ARKit system mainly adopts the hybrid tracking registration method combining Visual Inertial Odometry (VIO) and Inertial Odometry (IO). The VIO data are from the image information collected by the camera, and the IO data are from the Inertial Measurement Unit (IMU), including accelerometer and gyroscope, when the system is running; VIO and IMU calculate in parallel, and Kalman Filter or nonlinear optimization and other technical means are used for evaluating the two calculation results and select the better pose, to make the system obtain higher robustness [15]. AR real-time interaction behavior mainly aims at 6DOF control (degree of freedom) of 3D objects. The main interaction categories include touch-based interaction, air gesture-based interaction, and device-based interaction, while AR applications based on handheld mobile devices mainly interact by touching the screen to manipulate virtual objects or rotating devices around virtual objects. Virtual-real fusion refers to placing virtual objects into the real world and integrating them into one. ARKit framework is responsible for understanding the environment and transforming it into 3D scenes, and RealityKit is a new Swift rendering framework used for providing rendering support for ARKit [15]. RealityKit includes rendering, physical simulation, motion painting, 3D sound effect, and motion blur [15].

### 2.3. Overview of AR Experience Marketing

**Figure 2:**
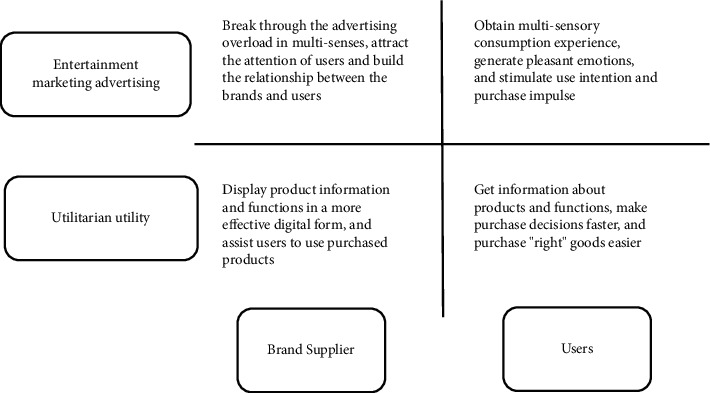
AR experience marketing application function matrix.

AR applications for experiential marketing are comprised of entertainment marketing advertising and utilitarian utilities. Entertainment AR marketing advertising tends to start from the technical characteristics of AR, increase multisensory stimulation for users from the dimension of virtual and real integration, prevent advertising information overload, build a communication and interaction bridge between users and brands from the real-time interaction dimension, and deepen the understanding of users about the brand improvement attitude; the utilitarian AR utility starts from the characteristics of different goods, fully displays product information and functions, improves purchase impulse and use intention of users, helps users make purchase decisions faster and more correctly, and optimizes consumption experience of users [16, 17]. The AR experience marketing matrix is shown in Figure 2.

#### 2.3.1. Entertainment AR Marketing Improves the Brand Attitude of Users

AR experience marketing impacts the psychology and emotion of users by strengthening their sensory stimulation, produces novel and beautiful feelings, enabling users to actively participate in interaction, independently explores the corporate culture and feels the brand concept, improves the act willingness, psychological feeling, and sense of value recognition of users, improves the brand impression of users, and builds a deep connection between users and consumers [18, 19]. Entertainment AR experience marketing is widely used in the IP promotion of cultural and tourism venues, such as scenic spots and museums. For example, the AR application of Malacca People's Museum is with many functions, such as virtual games, virtual kite making, and traditional beauty accessories [20]. Lin and Chen [21] designed an AR application and Q&A system for marketing tourist attractions in Thailand based on tourism brochures. Lin et al. [22] designed the identification of cultural relic cards and 3D coloring AR system to promote the new Zhongxin Village and promote the marketing of village communities to show cultural value and increase interaction with users. Boboc et al. [23] proved that AR application plays an important role in promoting intangible heritage, and VR application (including AR) technology can help tourism products publicize cultural information and enhance cultural influence, which has great application potential for improving the cultural tourism experience of users [24].

#### 2.3.2. Practical AR Marketing Improves the Purchase Intention of Users

The traditional marketing model only focuses on the many factors of products, such as packaging, function, and quality, but AR virtual experience marketing integrates the needs of users for actual experience, strengthens cognition of users on products from trial wearing, trial wearing, and other ways, provides users with detailed information and evaluation feedback of products [25], and improves the authenticity and reliability of marketing content. AR experience marketing can optimize the customer decision-making processes [26], enhance the consumption intention of users, and enable users to make purchase decisions more quickly and purchase the “right” goods [27, 28] through comparing with traditional marketing advertisements and traditional e-commerce platforms. AR experience marketing software is applied in different product fields as a utilitarian utility, such as clothing, real estate, beauty, food, and household appliances.

AR marketing tools are mostly used in clothing sales to provide users with a virtual fitting function [29]. For example, Xie et al. [30] designed the AR virtual fitting system to provide users new shopping experience. Chiu and Lee [31] added the functions of saving fitting pictures and shared on social platforms based on AR fitting. The AR fitting mirror scored higher in interface design, system availability, and interaction than the other two types of virtual fitting software on the market. AR marketing tools can help users try beauty products and wear accessories. For example, Ventes Avenues uses AR virtual fitting technology to design AR filters for Indian jewelry. Users can see the fitting effect through the filters without going to the store to try personally, providing users with an efficient and convenient shopping experience. AR marketing tools can display real estate information for users from a multidimensional and global perspective, helping users reduce decision-making time and improve purchase efficiency in real estate. For example, Mufid et al. [32] also designed an AR system to promote housing products and display the plans of different house types. 97.25% of the respondents were interested in this application. Furthermore, AR marketing tools can help machinery and household appliances to demonstrate the workflow and functional parameters. AR applications built by Udayan et al. [33] can show the name of valve products, product video links, and 3D models and key parameters. In home decoration, IKEA Place application supports users to select home products online and put the virtual home model into the real environment to watch the overall layout effect. In food commodities, Todorovic et al. [34] proposed that the application of AR in food packaging can display food supply chain and provide sufficient food information to users, including food attributes, place of origin, and production mode.

### 2.4. Common AR Marketing Evaluation Theory

Technology Acceptance Model (TAM) [21, 34, 35], mobility theory [35], use and satisfaction theory (UGT) [21], Stimulus-Organism-Response (S-O-R) [35, 36], and users experience theory [37] have been applied to the model construction of AR application and experience marketing. Davis proposed the Technology Acceptance Model (TAM) in 1989, which is used for predicting the acceptance of new digital technologies by users. It is the most widely used basis for AR application evaluation [38]. The S-O-R model covers the hedonic and utilitarian factors of users and reflects the core essence of AR technology from the perspective of users' experience [36]. User experience (UX) is defined as the “perception and response of users to use (or expected use) product or system,” including instrumental indicators, such as practicability and usability, and noninstrumental indicators, such as entertainment and attraction [37].

Schmidt first defined experience marketing systematically. He believes that experience marketing is a marketing model based on experience. The brand supplier takes products or services as the content carrier and uses artistic or technical means to bring public a new and beautiful marketing experience [1]. Schmitt proposed strategic experience modules according to the differences between the psychological cognitive process, cognitive connection, and experience media of users and divided experience marketing into five experience modules: sensory experience, emotional experience, thinking experience, action experience, and association experience [1].

## 3. Implementation of AR System for Museum Relic Card

**Figure 3:**
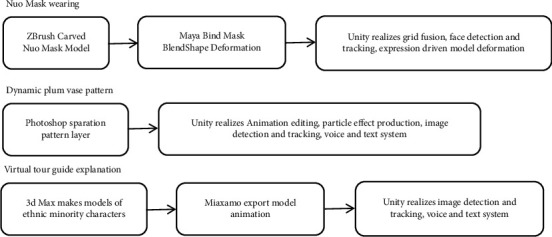
Technical roadmap of AR application in Guilin Museum.

The collection of Guilin Museum features the plum vase unearthed in Guilin in the Ming Dynasty, Guilin Historical Relics, and Guangxi minority cultural relics. According to official statistics, in the basic exhibition and special exhibition halls, 22% of the tourists liked the “Exhibition of the Guilin History and Culture,” 19% of the tourists liked the “Exhibition of the Guilin Folk Culture,” and 15% of the tourists liked the “Exhibition of the Plum Vase Unearthed in Guilin in the Ming Dynasty.” From the dimensions of users' cultural tendency and emotional preference, the distinctive cultural symbols in the museum are selected; that is, Nuo Mask, Plum Vase, and Custom Live Exhibition are used as the contents of cultural relic cards. The specific technical route of the AR system is shown in Figure 3.

### 3.1. AR Application Making about Nuo Mask Card

Nuo Mask is a special symbolic symbol. It shows the image and character of each mask in the opera through different facial features, completes the image shaping through the changes of facial features, and vividly shows the joys, sorrows, and joys of all characters. Therefore, this study focuses on the expression symbols of the Nuo Mask, makes the “Nuo Mask” 3D model with Maya, and uses the Blendshapes deformation technology used for the facial expression factor-driven model in the ARKit framework to control the deformation interaction effect of the expression-driven model.

#### 3.1.1. Expression Location of Nuo Mask Model of Blend Shape Location

**Figure 4:**
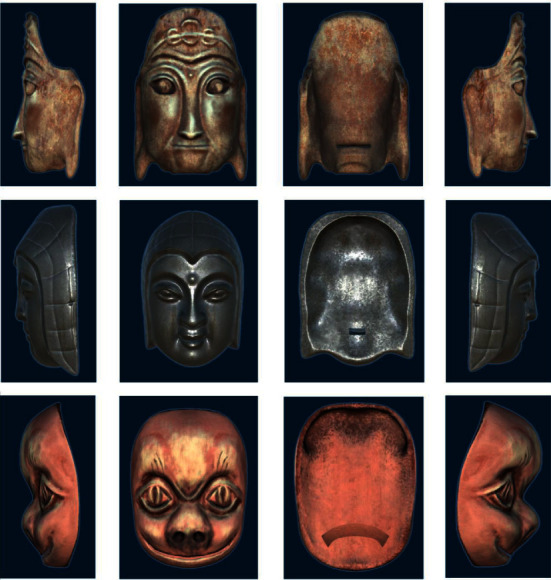
Nuo Mask model.

**Figure 5:**
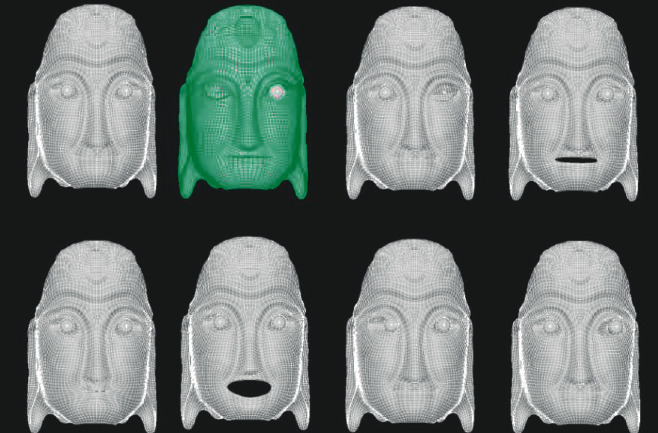
Nuo Mask deformation topological grid.

**Figure 6:**
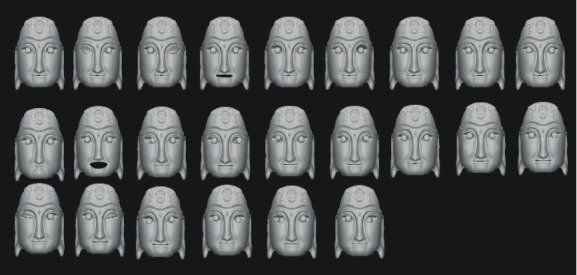
Set the animation of blend shape in Maya.

Blendshapes in ARKit refers to the technology of using a depth camera to collect users' facial expression features and driving model deformation with expression factors. It defines and stores the data dictionary of 52 expression feature motion factors in seven groups: left eye, right eye, mouth and chin, eyebrow, cheek, nose, and tongue. Firstly, the Nuo Mask model is carved in ZBrush software, and then the fusion deformation animation is made for the Nuo Mask model according to the positioning point of blend shape location in Maya software [15]. The Nuo Mask model is shown in Figure 4. And the topological mesh of the deformed mask model is shown in Figure 5. Use Maya's Blend Shape function to set the deformation animation as shown in Figure 6.

#### 3.1.2. Face Detection and Tracking

Face detection is a technology that uses computer graphics technology to locate the face in the figure, while face tracking aims to track the position and direction of the face. With the continuous development of deep neural network, face detection has become a more mature technology in the field of Artificial Intelligence (AI), and the accuracy has been significantly improved. AR Foundation realizes the function of detecting, tracking, and deforming the user's face with the help of the face detection algorithm and Blendshapes function provided by the underlying ARKit. ARKit regards the user's head as a 3D structure and first uses the Euler angle of the Cartesian left-hand coordinate system to locate the pose of the head contour, and the coordinate position is located at the tip of the nose; the system further detects the face features, which will be distributed on both sides of the face *Y*-axis [15].

**Figure 7:**
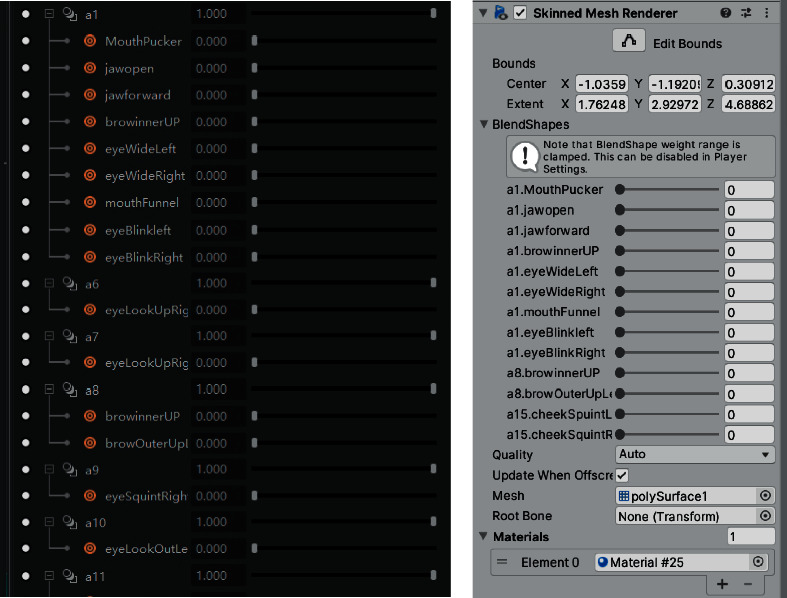
Deformation editing in Maya and mesh fusion of Blendshapes in Unity.

**Figure 8:**
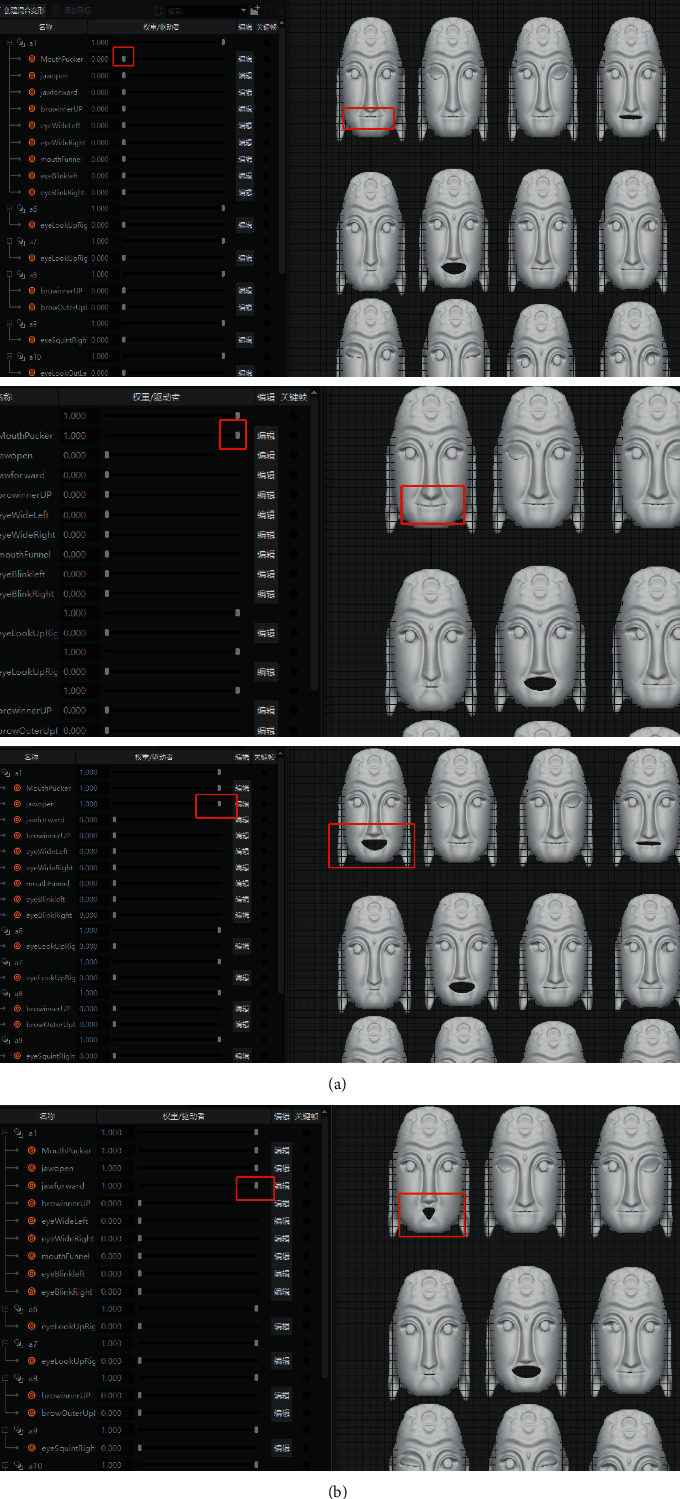
Mask model deformation under the influence of weight.

**Figure 9:**
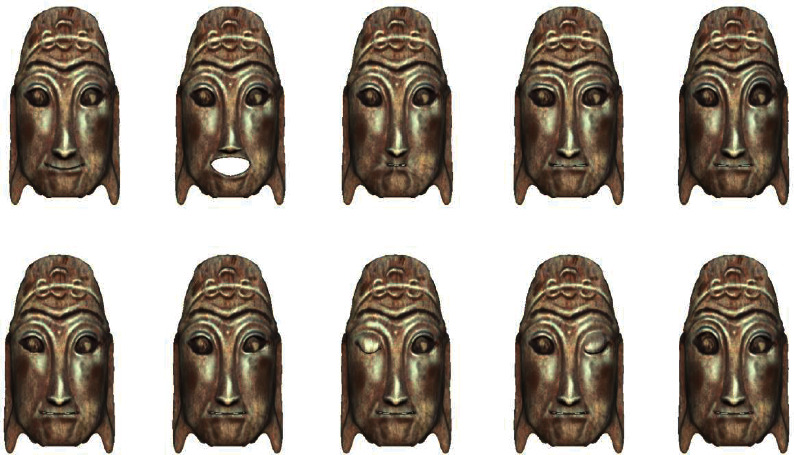
Nuo Mask model and deformation effect.

The bottom layer of ARKit provides AR Foundation with the user's face mesh detected by the system, including the vertex, index, normal, and texture coordinates of the face [15]. Developers shall use the set blend shape weight method to fuse the face mesh detected in the camera with the preset Nuo face model mesh, the network positioning points of Nuo Mask are corresponding to the blend shape location defined by Blendshapes, and the deformation weight is set according to the personality characteristics of different Nuo Mask [15]. Nuo Mask and users face have the same topology, but the vertex positions of different faces are different. The realization of deformation weight is to complete the filtering from Nuo Mask mesh to users face mesh. The following is the formula of weight and Nuo Mask model under the influence of different weight values [15]:

(1) $$\begin{array}{c} {\text{Value}}_{\text{fin}}=\left(1-\text{weight}\right){\times \text{Values}+\text{weight}\times \text{Value}}_{\text{t}}, \end{array}$$

where weight represents the weight value set by the system, value_fin_ represents the final grid fusion location value, value_s_ represents the source topology grid location data, and value_*t*_ represents the target topology grid location data. The work of implementing morphing editing in Maya and mesh fusion in Unity with Blendshapes is shown in Figure 7. The deformation effect of the mask model under the influence of weight is shown in Figure 8. And the final model and deformation effect of the Nuo Mask are shown in Figure 9.

**Figure 10:**
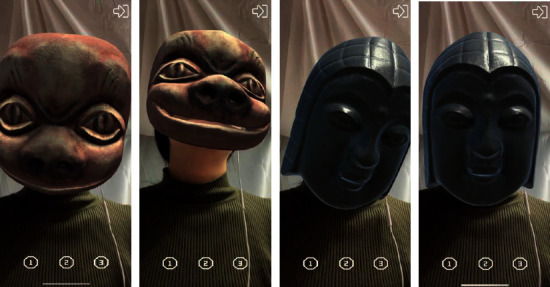
Wearing effect of ordinary Nuo Mask.

**Figure 11:**
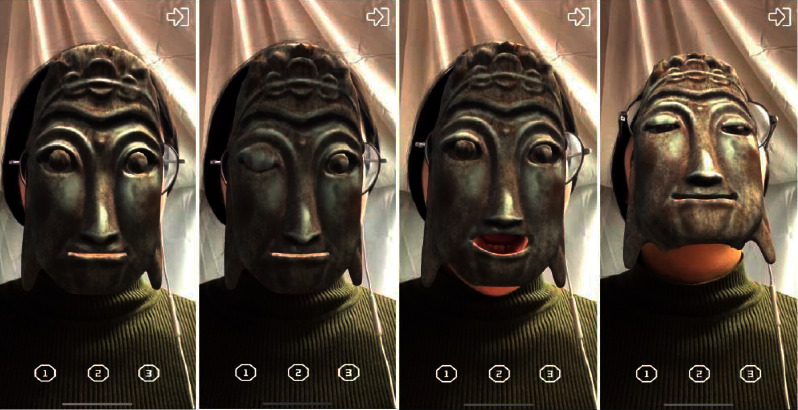
Wearing and morphing effect of Nuo Mask after adding Blendshapes.

Finally, Unity is used for completing the image recognition, virtual wearing, and deformation integration of cultural relic cards, and the AR module of “Nuo Mask” based on depth camera is developed. The following is the Nuo Mask model made according to the Nuo Mask in Guilin Museum and worn on the tourists' faces in real time. Some masks have the function of face-driven deformation of Blendshapes, and the mask shape can be controlled through expression. The wearing effect of the ordinary Nuo Mask is shown in Figure 10, and the wearing effect of the transformable Nuo Mask is shown in Figure 11.

### 3.2. AR Application Making about Dioramas Card and Plum Vase Card

Image detection is a technology to recognize, locate, track, and register the 2D image in the camera through a computer graphics algorithm. When the feature values of the image corresponding to the image stored in the system are prematched to the feature values of the image stored in the system, the AR system can recognize plum vase pictures by scanning plum vase cards and display plum vase pattern animation and text explanation, accompanied by voice explanation; scanning the real scene card can identify the image of the real scene of the exhibition, and the virtual commentator dressed in minority costumes can appear in the collection screen to explain vividly with different actions. The core code of real-time detection and updating of cultural relic cards is shown in Table 1.

#### 3.2.1. Making of Dioramas Card

**Figure 12:**
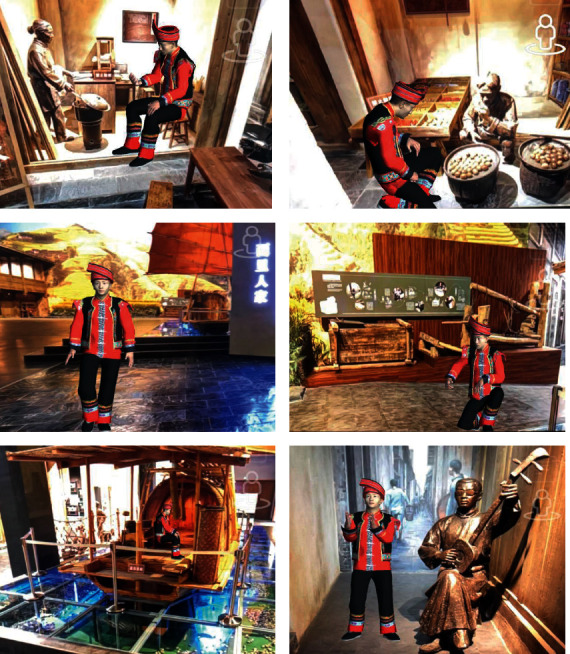
AR display effect of virtual commentator.

According to the folk customs carried by the exhibition of dioramas, the model animation of the virtual commentator is designed and bound to the corresponding dioramas card. The users can scan the dioramas card to watch the explanation of the virtual commentator. The demonstration effect is shown in Figure 12.

#### 3.2.2. Making of Plum Vase Card

**Figure 13:**
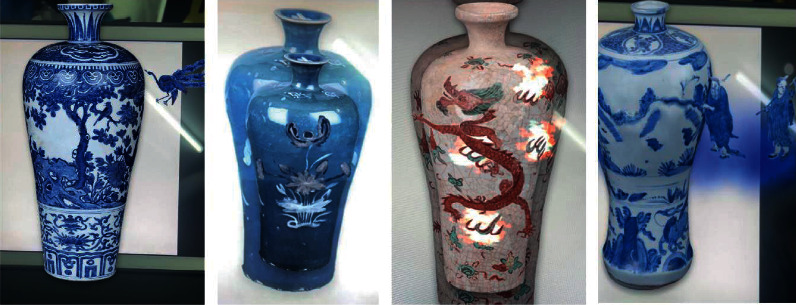
AR display effect of dynamic plum vase pattern.

The characteristics of plum vase patterns are analyzed through plum vase data, design dynamic effects according to the cultural connotation carried by the patterns, and properly add particle effects using a particle system. For example, aiming at the main pattern of “Blue and White Plum Vase with Hundreds Birds Worshipping the Phoenic,” the dynamic effects of birds flying were made; aiming at the main pattern of “Blue Glaze Overgalze Coluored Plum Vase with Egret and Cyan Lotus,” the dynamic effects of blooming and swaying lotus flowers were created, and the particle effects of “flowing water in the pool” were added; aiming at the main pattern of “Ge Glaze Plum Vase with Five-colored Cloud-Dragon,” the dynamic effects of swinging dragon and the particle effects of flame burning were designed; the main pattern of “Blue and White Plum Vase with Eight Immortals” is characters of the Eight Immortals which was widely popular in the Ming Dynasty, so the dynamic effects of the immortals and the particle effects of them coming from the clouds were made for this plum vase. The demonstration effect is shown in Figure 13.

## 4. Propose Assumptions and Build Models

Technology application, need, and experience quality shall also be considered in AR virtual experience marketing model, and Schmitt strategic experience module is combined with other common AR evaluation modules. Finally, the following variables and assumptions are proposed from the two perspectives of AR technology dimension evaluation indicators and dimension experience indicators of users based on the Schmitt strategic experience module:

1. *Sensory Experience*. Sensory experience refers to the perceptual experience generated by users while being stimulated by external senses. Sensory experience is mainly comprised of three evaluation indexes: technical usability, sensory stimulation, and sensory immersion. From the perspective of AR digital application marketing, a virtual digital experience is created to let users understand product details or relevant functional information through vision, hearing, taste, and touch, provide the users sensory stimulation, make them be excited and satisfied, expand the added value of products, and cause behavior tendency and purchase desire of users. For example, AR shows 3D models and image animation related to brand culture on-screen, which has attracted strong attention of users from the visual and auditory levels.
2. *Emotional Experience*. With the development of digital age and experience economy, there are many factors impacting the psychological emotion of users. Brands shall use AR to condense brand connotation and product culture into a virtual experience, stimulate the inner emotion of users to resonate, create a beautiful and novel experience for them, and shorten the emotional distance between users and brands and products. Emotional experience takes “whether the application is interesting,” “whether it produces emotional resonance,” and “whether it produces positive emotion” as the evaluation indicators. Groenroos and Voima [39] pointed out that “emotional resonance” is generated from the consistency between the psychological state of users and the information provided by the system, including expectation consistency, value participation, and spiritual harmony.
3. *Thinking Experience*. By understanding the cognitive system and interest tendency of users, brand suppliers provide relevant information about the product through AR marketing application, show the performance function of the product, appropriately eliminate unknown risks, help users make decisions, guide users to think, stimulate their creative thinking, enable users to deeply understand the brand and products and gain profound cognition, and arouse use intention and purchase tendency of users. Thinking experience is based on the dynamic metacognition process of users, takes the degree of information and usefulness, and triggers thinking and knowledge retention of users as the evaluation indicators [40, 41]. Among them, Lam et al. [40] confirmed that AR application can help users acquire or retain new knowledge through pretest and posttest experiments.
4. *Action Experience*. In AR marketing, the action experience module refers to the interactive behavior between users and virtual elements. AR applications design interactive forms according to users' needs to strengthen users' experience [42] and improve their sense of participation to actively establish the relationship between brands and users. Behavior feedback of users also improves their metacognition of brands and products continuously. The action experience takes the form of immediate interaction, interaction form, and participation enthusiasm as three measurement indicators.
5. *Associated Experience*. Relevance experience is based on the comprehensive consideration of the sociological dimension. In AR application, it refers to the interaction between users and others, interaction with social networks, recommendation of the application to others, and then creating a consistent atmosphere through the evaluation and recognition of the brand to build a good public opinion atmosphere and consumption situation.
6. *Brand Attitude*. The form of AR experience marketing can provide users with strong sensory stimulation, positive pleasant emotion, practical functional information, and innovative real-time interaction and even build social networks around virtual experience. Using AR system can enable users to obtain a satisfactory experience and produce a positive brand impression and high brand loyalty [43]. Compared with traditional advertising methods, users of AR marketing advertising have a 200% higher memory of brands, products, and functions [33]. Furthermore, based on the application of mixed reality in cultural and tourism attractions, Bae et al. [44] confirmed that the characteristics of mixed AR technology have a positive impact on experience satisfaction and brand loyalty.
7. *Use Intention and Purchase Intention*. After users experience AR marketing advertisements or AR marketing tools, their intention to use or make decisions about purchase is a comprehensive evaluation based on product information, brand impression, and experience quality [45]. Furthermore, Huang and Liu [46] and Haile and Kang [47] proposed that the AR system has a certain persuasive effect, which also impacts the judgment and behaviors of users.

Structural equation model is a statistical method used for analyzing the relationship between variables based on the covariance matrix of variables. It is an important tool for multivariate data analysis. According to the above analysis indicators, assumptions are proposed, and the structural equation model is constructed as follows:

  H1: Sensory experience positively impacts brand attitude of users.
  H2: Emotional experience positively impacts brand attitude of users.
  H3: Thinking experience positively impacts brand attitude of users.
  H4: Action experience positively impacts brand attitude of users.
  H5: Association experience positively impacts brand attitude of users.
  H6: Sensory experience improves use intention and purchase intention of users.
  H7: Emotional experience improves use intention and purchase intention of users.
  H8: Thinking experience improves use intention and purchase intention of users.
  H9: Action experience improves use intention and purchase intention of users.
  H10: Association experience improves use intention and purchase intention of users.
  H11: Brand attitude of users impacts use intention and purchase intention of users.

**Figure 14:**
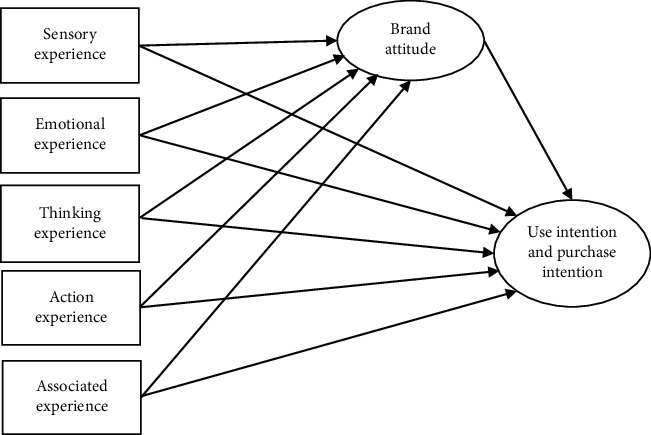
AR virtual experience marketing structure model.

The structure model of AR virtual experience marketing is constructed as shown in Figure 14. The variables, elements, item settings (for Guilin Museum cultural relics card), and references of the AR Virtual experience strategic model are shown in Table 2.

## 5. Empirical Study on AR Virtual Experience Marketing

A questionnaire survey was conducted on the public by random sampling herein. Before making the questionnaire, each respondent was given more than 10 cultural relics cards (including Nuo Mask card, plum vase card, and real scene card) and an iPhone equipped with the Guilin Museum AR Application. The respondents could wear a virtual Nuo Mask by scanning the Nuo Mask card with Guilin Museum AR Application and try facial expression-driven Nuo Mask, which had the interactive function of deformation. Scanning plum vase cards, the users could see vivid plum vase pattern animation, scanning real-life exhibition pictures, they could see virtual commentators dressed in ethnic minority costumes, and each card was accompanied by text description and audio explanation. After the users had experienced the AR application of Guilin Museum, the respondents were asked to fill in the basic information and questionnaire. The complete study time was about 15 min.

The questionnaire method was used to verify the study questions herein. The paper questionnaire contains 7 factors and 21 measurement items, including 3 sensory experiences, 3 emotional experiences, 3 thinking experiences, 3 action experiences, 3 association experiences, 3 brand attitudes, and 3 use and purchase intentions, all of which were measured by Likert 5-point scale (i.e., 1-very disagree, 2-disagree, 3-general, 4-agree, and 5-very agree), and participants have the right to decide whether to fill in the questionnaire. The questionnaire mainly includes two parts: the first part aims to collect the basic information of participants, including gender, age, and education; the second part aims to collect the measurement scale of hypotheses h1–h11.

### 5.1. Interviewee Characteristics

In the public random sampling survey, 276 volumes were distributed, and the samples that did not meet the standards were excluded. The effective sample size of the questionnaire was 219. Since the autonomy of participating in AR application test and questionnaire filling completely depends on street respondents, the statistical data of the respondents is shown in Table 3 and it could be seen from the table that males account for 58.90% and females account for 41.10%. Males may be more willing to try new technologies. For example, Wakim et al. [57] also confirmed that males were more attracted by AR technology in the study; meanwhile, the proportion of teenagers under 25 was as high as 61.19%. It can be seen that the AR experience effect is more attractive to young people, who highly accept new technology. Zhang et al. [51] mentioned that users at different ages have different attitudes towards this technology.

### 5.2. Evaluation of Measurement Model

Reliability analysis is the test of the stability, consistency, and reliability of the measurement results. In order to ensure the accuracy of the overall measurement results of the questionnaire, Cronbach's *α* coefficient was adopted for analyzing seven indicators in the questionnaire, including sensory experience, emotional experience, thinking experience, action experience, associated experience, brand attitude, and use intention and purchase intention. The results of the reliability analysis are shown in Table 4. And the reliability of each index is higher than 0.85, proving that the data of this questionnaire is reliable. Validity refers to the validity of the measurement purpose in the questionnaire, in which KMO value of simple correlation and partial correlation coefficient between items is higher than 0.7, which is suitable for factor analysis; if the significance of the Bartlett spherical test is 0.001 < 0.01, the validity of the data is good with a significant relationship between various indexes. The results of the validity analysis are shown in Table 5.

The principal component analysis method is used for extracting the factors of each index data, and a total of 6 common factors are extracted. The results of total variance interpretation are shown in Table 6. The cumulative variance contribution rate is 78.748%, higher than 60%. The interpretation degree of the extraction is high, indicating that the effect of the extracted factors is good.

### 5.3. Correlation Analysis

Correlation analysis refers to the process of describing and analyzing the nature and degree of correlation of the relationship between two or more variables, and the analysis results are shown in Table 7. The correlation coefficient in the upper right corner with mark ^*∗*^ indicates that there is a relationship; otherwise, there is no relationship. When the correlation coefficient is >0, this indicates that there is a positive correlation between the two variables, and when <0, this indicates that there is a negative correlation between the two variables. ^*∗*^ represents being at the level of 0.05 (double tail), and the correlation is significant. Therefore, it can be seen from Table 7 that there are significant positive correlations among all the items.

### 5.4. Structural Equation Model

**Figure 15:**
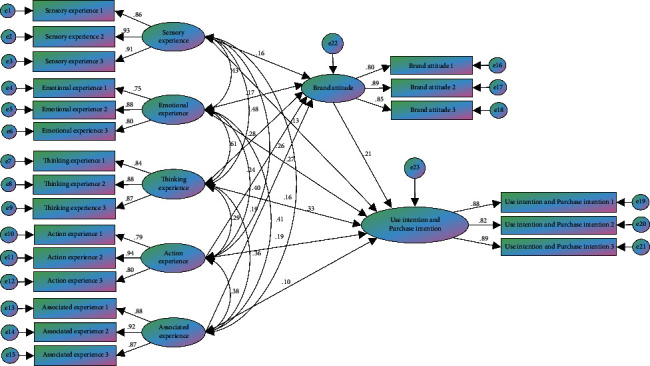
Structural equation model.

AMOS software is used for calculating various parameter indexes in the model and testing the significance of various indexes. The structural equation model (SEM) adopts the two-step method of Anderson and Gerbing [58]. In the first-order confirmatory factor analysis model, if the potential variables have a correlation and the correlation is >0.6, the second-order confirmatory factor could be analyzed. It can be seen from Table 8 that, through the analysis of the model fitting, the Chi-square degree of freedom ratio of the confirmatory factor analysis model herein is <3, the square root RMSEA of the approximation error is 0.043, and most of the other indicators have good fitting effects. The model is established as shown in Figure 15.

Through further analysis of the structural model, the path coefficient diagram of sensory experience, emotional experience, thinking experience, action experience, and associated experience on brand attitude and use intention and purchase intention are obtained, as shown in Table 9. According to the statistical data analysis, the standardized path coefficient of sensory experience on brand attitude is 0.165, *p* < 0.05, indicating that there is a significant positive impact relationship, and the hypothesis is tenable; the standardized path coefficient of sensory experience on use intention and purchase intention is 0.134, *p* < 0.05, indicating that there is a significant positive impact relationship, and the hypothesis is tenable. Sensory experience is used to strengthen sensory stimulation and attract users' attention. It has been proved to have significant impacts on brand attitude and use intention and purchase intention. The sensory stimulation factors in AR experience marketing have been realized herein through the 3D model of Nuo Mask, dynamic animation of plum vase pattern, virtual tour guide, and voice explanation in the museum. The study shows that AR application can use the senses of vision, hearing, and touch to create a pleasant virtual sensory experience for users by promoting brand culture and demonstrating product functions, to arouse the interest and attention of users. Meanwhile, the better the incentives are, the stronger the users' impression of brand culture and use intention and purchase intention will be.

The standardized path coefficient of emotional experience on brand attitude is 0.165, *p* < 0.05, indicating that there is a significant positive impact relationship, and the hypothesis is tenable; the standardized path coefficient of emotional experience on use intention and purchase intention is 0.266, *p* < 0.05, indicating that there is a significant positive impact relationship, and the hypothesis is tenable. Emotional experience is used for tapping the individual and emotional needs of users, mobilizing the positive emotions of users, achieving spiritual harmony, and producing emotional resonance. It has been proved to have significant impacts on brand attitude and use intention and purchase intention. The interest and preference of museum tourists were selected for folk history in this paper, and the study proves that creating an emotional experience for the users and excavating the internal feelings and emotions of users can make users have a positive brand attitude culture.

The standardized path coefficient of thinking experience on brand attitude is 0.276, *p* < 0.05, indicating that there is a significant positive impact relationship, and the hypothesis is tenable; the standardized path coefficient of thinking experience on use intention and purchase intention is 0.162, *p* < 0.05, indicating that there is a significant positive impact relationship, and the hypothesis is tenable. Thinking experience has been proved to have significant impacts on brand attitude and use intention and purchase intention, which are reflected in the process of human cognition. In the early stage, it provides users with basic information about the product, displays functional characteristics, meets the information needs of users, continuously guides users to think about the processes, and significantly improves knowledge retention and brand impression of users on products. The study shows that the creative way can strengthen users' thinking ability about problems. AR virtual experience can make products transition from offline to online. Virtual information can meet the display needs of different types of products, such as displaying the production chain of foods, the trial wearing effect of jewelry, and the function of products, to improve the authenticity of products and the credibility of brands.

The standardized path coefficient of action experience on brand attitude is 0.242, *p* < 0.05, indicating that there is a significant positive impact relationship, and the hypothesis is tenable; the standardized path coefficient of action experience on use intention and purchase intention is 0.189, *p* < 0.05, indicating that there is a significant positive impact relationship, and the hypothesis is tenable. Action experience has significant impacts on brand attitude and use intention and purchase intention. In AR marketing application, meaningful interaction behaviors shall be designed to make users unconsciously enter the flow state and improve the quality of experience interaction. For example, this study makes users unconsciously mobilize their five senses to interact through the experience effect of face interaction model, strengthen the connection with products, improve the impression of the brand, and cause users' impulsive behaviors through interaction with products [35].

The standardized path coefficient of associated experience on brand attitude is 0.185, *p* < 0.05, indicating that it shows a significant positive impact relationship, and the hypothesis is tenable; the standardized path coefficient of associated experience on use intention and purchase intention is 0.096, *p* > 0.05, indicating that there is no significant impact relationship, and the hypothesis is not tenable. Relevance experience has significant positive impacts on brand attitude, but nonsignificant direct impact on use intention and purchase intention. This hypothesis is not tenable. However, the AR marketing advertisement used for brand promotion can create links with family, peers, and social platforms, with a good effect on improving the cognitive depth and breadth of the brand and building the brand atmosphere.

## 6. Conclusion and Future Work

In current electronic consumption times, marketing is not limited to product sales, but it can be used to build the connection between Brand IP Culture and users from the macrolevel, show the information, functions, and ideas of products to the users from all dimensions, and regard users as a combination of rationality and sensibility to meet the personalized needs of users in the new era. Based on the perspective of experiential marketing, this paper chooses Unity as the software development tool and AR Foundation as the AR development framework to develop an innovative AR application for promoting Guilin Museum. The application uses technologies such as face detection and tracking, image detection and tracking, and Blendshapes model deformation in ARKit. This paper selects Nuo Masks, plum vases, and real-life exhibition halls in the museum as the main content of the cultural relic card. When users scan the Nuo Mask card with the AR application, they can wear the 3D virtual Nuo Mask on face and drive the deformation of the Nuo Mask through facial expressions; when the user scans the plum vase card, they can see the character animation and particle effect of the static plum vase pattern; when the user scans the real scene card, they can see the different forms of ethnic minority virtual guides explaining the scenic spots.

In addition, another contribution of this paper is to build an AR virtual experience marketing model based on Schmitt's experience strategy model and use the AR application of Guilin Museum to conduct an empirical analysis of the model. The study results show that the traditional marketing model regards the users as a reasonable embodiment, but experience marketing combines the rationality and sensibility of users. AR virtual experience marketing makes users involuntarily participate through the technical characteristics of VR integration and real-time interaction, arouses the curiosity of users through sensory stimulation, and resonates with the users through emotional projection through mobilizing users' thinking to make them understand the brand and products, through action guidance to make users unconsciously interact with the brand and products, and make users produce perceptual impulse by using community relations and enhancing shared value. This kind of correlation behavior has no significant impact on the final purchase decision of users.

This paper only develops the museum AR application to evaluate the AR virtual experience model, but the promotion and marketing evaluation focus of different industries is different, so this paper still has certain limitations in case selection. Future work will focus on different types of brands and products, study their product characteristics and user needs to develop AR applications, and further verify the AR virtual experience marketing model to find the commonalities and differences between different types of products when using AR applications for marketing, deepening the depth and breadth of theoretical research.

## Figures and Tables

**Table 1:** Core code of real-time detection and updating of cultural relic cards.

private void TrackedImages (ARTrackedImagesChangedEventArgs eventArgs) {
foreach (var trackedImage in eventArgs.added){
AddedList (trackedImage);}
for (int *i* = 0; *i* < eventArgs.updated.Count; *i*++){
UpdatedImagesChanged (eventArgs.updated [*i*]);}
for (int *i* = 0; *i* < eventArgs.removed.Count; *i*++){
RemovedList (eventArgs.removed [*i*]);}}
private void UpdatedImagesChanged (ARTrackedImage referenceImage){
string targetImage = referenceImage.referenceImage.name;
models[targetImage]?.SetActive (referenceImage.trackingState = = TrackingState.Tracking);
SetIntroduce(referenceImage);
AudioClip audioClip = GetAudioClip (clipDic, referenceImage);
PlaySound (audioSource,audioClip);}

**Table 2:** AR virtual experience strategic model variables, elements, item settings (for Guilin Museum cultural relics card), and references.

Model variables	Essential factor	Measurement item	Reference
Sensory experience	Technical usability	AR application can be used more easily	Lin and Chen [21], Boboc et al. [23], Do et al. [35], Qin et al. [36], Morillo et al. [48], and Adrianto et al. [49]
	Sensory irritation	AR application has positively stimulated my senses (including vision, hearing, taste, smell, and touch), and I have a certain perception of cultural relics	
	Sense immersion	AR application allows virtual objects to better integrate into the real world. I am satisfied with the rendering effect and enjoy it	

Emotional experience	Interesting application	AR application has aroused my interest and curiosity about museum relics	Brito et al. [18], Siang et al. [20], Lin and Chen [21], Qin et al. [36], Morillo et al. [48], Poitras [50], Zhang et al. [51], Smink et al. [52], Kang [53], and Li and Fang [54]
	Emotional resonance	Agree with the cultural value and folk spirit conveyed by AR application	
	Positive emotions	AR application makes me feel curious, happy, excited, and other positive emotions	

Thinking experience	Information and usefulness	The AR application helps me to have an in-depth understanding of museum cultural relics and meets my needs for information cognition. AR system is considered useful	Lu and Smith [27], Udayan et al. [33], Qin et al. [36], Zhang et al. [51], Smink et al. [52], and Kang [53]
	Trigger thinking	AR application allows me to think more deeply and actively about cultural relics	
	Knowledge retention	Compared with the traditional text interpretation form, with deeper understanding of the virtual information provided by AR application and more knowledge retention	

Action experience	Instant interaction	The interaction of AR system is immediate and effective	Brito et al. [18], Do et al. [35], Qin et al. [36], Morillo et al. [48], and Huang and Liao [55]
	Interactive form	The interaction form of AR system is more interesting	
	Active participation	AR system makes me want to actively experience and participate in it, with a high degree of participation	

Associated experience	Associate with peers	Hope to experience the AR effect with my friends around me	Lin and Chen [21] and Chiu and Lee [31]
	Associated with platform	Hope to share the experience effect on the social platform	
	Application promotion	Hope to recommend the AR app to others	

Brand attitude	Experience satisfaction	Satisfied with the experience of AR application in Guilin Museum	Brito et al. [18], Lin and Chen [21], Qin et al. [36], Bae et al. [44], Zhang et al. [51], Smink et al. [52], and Cruz et al. [56]
	Brand impression	A positive and good impression is left in the Guilin Museum	
	Brand loyalty	Compared with other similar museums, give priority to the Guilin Museum	

Use intention and purchase intention	Persuasive effect	After using the AR application, the idea of deeply understanding the Guilin Museum was generated. Before that, only cultural relic cards were watched, without this idea	Lin and Chen [21], Grewal et al. [26], Wang et al. [28], Do et al. [35], Qin et al. [36], Huang and Liu [46], Haile and Kang [47], Smink et al. [52], and Kang [53]
	Use intention	Want to use AR system to visit the Guilin Museum	
	Purchase intention	Want to purchase museum cultural relic cards or visit Guilin Museum in person	

**Table 3:** Demographic data of respondents.

Variable	Classification	Number of cases	Percentage
Gender	Male	129	58.90
	Female	90	41.10

Age	<18	18	8.22
	18–25	116	52.97
	25–35	32	14.61
	35–45	37	16.89
	45–55	16	7.31

Education	Primary school	13	5.94
	Junior high school	28	12.79
	High school	30	13.70
	Undergraduate college	109	49.77
	Graduate school	39	17.80

**Table 4:** Reliability analysis of questionnaire.

Gauge	Cronbach's coefficient *α*	Number of items
Sensory experience	0.926	3
Emotional experience	0.850	3
Thinking experience	0.895	3
Action experience	0.874	3
Associated experience	0.921	3
Brand attitude	0.883	3
Use intention and purchase intention	0.897	3

**Table 5:** Validity analysis of questionnaire.

KMO value		0.889
Bartlett spherical test	Approximate Chi-square	3447.166
	Freedom	210
	Significance	0.001

**Table 6:** Interpretation of total variance.

Component	Initial eigenvalue			Extract the sum of squares of loads			Sum of squares of rotating loads		
	Total	Percentage variance	Cumulative%	Total	Percentage variance	Cumulative%	Total	Percentage variance	Cumulative%
1	8.997	42.841	42.841	8.997	42.841	42.841	3.249	15.469	15.469
2	2.105	10.024	52.866	2.105	10.024	52.866	2.813	13.396	28.865
3	1.686	8.028	60.894	1.686	8.028	60.894	2.736	13.029	41.894
4	1.540	7.332	68.226	1.540	7.332	68.226	2.668	12.703	54.596
5	1.175	5.594	73.819	1.175	5.594	73.819	2.573	12.254	66.850
6	1.035	4.929	78.748	1.035	4.929	78.748	2.499	11.898	78.748
7	0.944	4.494	83.242						
8	0.457	2.176	85.418						
9	0.393	1.870	87.287						
10	0.337	1.605	88.893						
11	0.331	1.578	90.470						
12	0.298	1.419	91.890						
13	0.267	1.270	93.159						
14	0.246	1.172	94.332						
15	0.238	1.133	95.465						
16	0.199	0.948	96.413						
17	0.184	0.875	97.287						
18	0.179	0.851	98.138						
19	0.146	0.694	98.832						
20	0.126	0.598	99.431						
21	0.120	0.569	100.000						

**Table 7:** Correlation between factors.

	Sensory experience	Emotional experience	Thinking experience	Action experience	Associated experience	Brand attitude	Use intention and purchase intention
Sensory experience	1	0.374^*∗∗*^	0.451^*∗∗*^	0.258^*∗∗*^	0.306^*∗∗*^	0.441^*∗∗*^	0.471^*∗∗*^
Emotional experience	0.374^*∗∗*^	1	0.525^*∗∗*^	0.382^*∗∗*^	0.364^*∗∗*^	0.500^*∗∗*^	0.580^*∗∗*^
Thinking experience	0.451^*∗∗*^	0.525^*∗∗*^	1	0.278^*∗∗*^	0.329^*∗∗*^	0.525^*∗∗*^	0.539^*∗∗*^
Action experience	0.258^*∗∗*^	0.382^*∗∗*^	0.278^*∗∗*^	1	0.358^*∗∗*^	0.452^*∗∗*^	0.480^*∗∗*^
Associated experience	0.306^*∗∗*^	0.364^*∗∗*^	0.329^*∗∗*^	0.358^*∗∗*^	1	0.460^*∗∗*^	0.455^*∗∗*^
Brand attitude	0.441^*∗∗*^	0.500^*∗∗*^	0.525^*∗∗*^	0.452^*∗∗*^	0.460^*∗∗*^	1	0.598^*∗∗*^
Use intention and purchase intention	0.471^*∗∗*^	0.580^*∗∗*^	0.539^*∗∗*^	0.480^*∗∗*^	0.455^*∗∗*^	0.598^*∗∗*^	1

**Table 8:** Model fitting index.

Common indicators	*χ* ^2^/DF	RMSEA	GFI	NFI	RFI	IFI	TLI	CFI
Judgment standard value	<3	<0.10	>0.9	>0.9	>0.9	>0.9	>0.9	>0.9
	1.408	0.043	0.912	0.934	0.917	0.980	0.975	0.980

**Table 9:** Path coefficient diagram of the structural model.

			Standardization coefficient	SE	CR	*P*
Brand attitude	<-	Sensory experience	0.165	0.050	2.510	0.012
Brand attitude	<-	Emotional experience	0.165	0.085	2.017	0.044
Brand attitude	<-	Thinking experience	0.276	0.082	3.460	^*∗∗∗*^
Brand attitude	<-	Action experience	0.242	0.056	3.731	^*∗∗∗*^
Brand attitude	<-	Associated experience	0.185	0.056	2.858	0.004
Use intention and purchase intention	<-	Sensory experience	0.134	0.047	2.158	0.031
Use intention and purchase intention	<-	Emotional experience	0.266	0.083	3.386	^*∗∗∗*^
Use intention and purchase intention	<-	Thinking experience	0.162	0.080	2.097	0.036
Use intention and purchase intention	<-	Action experience	0.189	0.055	2.996	0.003
Use intention and purchase intention	<-	Associated experience	0.096	0.054	1.561	0.118
Use intention and purchase intention	<-	Brand attitude	0.208	0.085	2.465	0.014

## Data Availability

The data used to support the findings of this study are available from the corresponding author upon request.

## References

[1] Schmitt B. H. (1999). *Experiential Marketing: How to Get Customers to Sense*.

[2] Hsiao C. H., Yang C. C. Exploring the effect of experiential marketing on movie-watching intention--the example of mobile movie theme games.

[3] Huang J. S., Wei T. Social visibility and percept differentiation dimensions of brand intangible value and its effects.

[4] Liu L., Liu J., Lang J. An empirical study of consumer satisfaction based on experiential marketing of communications enterprises.

[5] Li T., Chen L. Research on operational strategies of B-to-C websites based on experiential marketing.

[6] Liang Y. P. The relationship between consumer experience, perceived value and impulsive buying behavior.

[7] Alimamy S., Deans K. R., Gnoth J. (2017). Augmented reality: uses and future considerations in marketing. *Leadership, Innovation and Entrepreneurship as Driving Forces of the Global Economy*.

[8] Milgram P., Kishino F. (1994). A taxonomy of mixed reality visual displays. *IEICE - Transactions on Info and Systems*.

[9] Azuma R. T. (1997). A survey of augmented reality. *Presence: Teleoperators and Virtual Environments*.

[10] Kang B. (2006). Tracking technology of augmented reality. *Computer Measurement & Control*.

[11] Han Y. R., Li T. J., Yang D. (2019). Overview of 3D tracking registration technology in augmented reality. *Computer Engineering and Applications*.

[12] Goh E. S., Sunar M. S., Ismail A. W. (2019). 3D object manipulation techniques in handheld mobile augmented reality interface: a review. *IEEE Access*.

[13] Yu J. D. (2018). *Design and Implementation of Museum Guide System Based on mobile Augmented Reality*.

[14] Long Z. S. (2020). *Design and Implementation of Museum Cultural Relic Display System Based on Augmented Reality*.

[15] Wang X. C. (2020). *Road to AR Foundation - AR Development from Beginning to Practice*.

[16] Javornik A. [Poster] classifications of augmented reality uses in marketing.

[17] Ross H. F., Harrison T. Augmented reality apparel: an appraisal of consumer knowledge, attitude and behavioral intentions.

[18] Brito P. Q., Stoyanova J., Coelho A. (2018). Augmented reality versus conventional interface: is there any difference in effectiveness?. *Multimedia Tools and Applications*.

[19] Choi H.-H., Lim S.-A., Jeong C.-S. (2016). New promotional video technique utilizing augmented reality and popcode. *Multimedia Tools and Applications*.

[20] Siang T. G., Ab Aziz K. B., Ahmad Z. B., Suhaifi S. B. Augmented reality mobile application for museum: a technology acceptance study.

[21] Lin H.-F., Chen C.-H. (2017). Combining the technology acceptance model and uses and gratifications theory to examine the usage behavior of an augmented reality tour-sharing application. *Symmetry*.

[22] Lin C. M., Lin T. C., Lin Y. C., Wang C.-M., Dow C. R. Community interaction and marketing using 3D coloring augmented reality in zhongxing new village.

[23] Boboc R., Duguleană M., Voinea G.-D. (2019). Mobile augmented reality for cultural heritage: following the footsteps of Ovid among different locations in Europe. *Sustainability*.

[24] González-Rodríguez M. R., Díaz-Fernández M. C., Pino-Mejías M. Á. (2020). The impact of virtual reality technology on tourists’ experience: a textual data analysis. *Soft Computing*.

[25] Arjun G. Pseudo Eye: the next-generation shopping application using Augmented Reality.

[26] Grewal D., Roggeveen A. L., Nordflt J. (2017). The future of retailing. *Journal of Retailing*.

[27] Lu Y., Smith S. Augmented reality E-commerce assistant system: designing while shopping.

[28] Wang C. H., Chiang Y. C., Wang M. J. (2015). Evaluation of an augmented reality embedded on-line shopping system. *Procedia Manufacturing*.

[29] Wang W. C., Chen Y. H., Kao C. Y. Integrating augmented reality into female hairstyle try-on experience.

[30] Xie B., Ye F., Li X. J. (2011). Research and application of virtual dressing system based on AR. *Advanced Materials Research*.

[31] Chiu C. C., Lee L. C. (2018). Empirical study of the usability and interactivity of an augmented-reality dressing mirror[J]. *Microsystem Technologies*.

[32] Mufid M. R., Tahir M., Meiyuana D. B., Dwileksa A. B. Housing design in planet green tambora using augmented reality for promotion media.

[33] Udayan J. D., Kataria G., Yadav R., Kothari S. Augmented reality in brand building and marketing–valves industry.

[34] Todorovic V., Milic N., Lazarevic M. Augmented Reality in Food production traceability–use case.

[35] Do H. N., Shih W., Ha Q. A. (2020). Effects of mobile augmented reality apps on impulse buying behavior: an investigation in the tourism field. *Heliyon*.

[36] Qin H., Peak D. A., Prybutok V. (2021). A virtual market in your pocket: how does mobile augmented reality (MAR) influence consumer decision making?. *Journal of Retailing and Consumer Services*.

[37] Olsson T., Salo M. Online user survey on current mobile augmented reality applications.

[38] Davis F. D. (1989). Perceived usefulness, perceived ease of use, and user acceptance of information technology. *MIS Quarterly*.

[39] Groenroos C., Voima P. (2013). Critical service logic: making sense of value creation and co-creation. *Journal of the Academy of Marketing Science*.

[40] Lam M. C., Sadik M. J., Elias N. F. (2021). The effect of paper-based manual and stereoscopic-based mobile augmented reality systems on knowledge retention. *Virtual Reality*.

[41] Zhang H., Cui Y., Shan H., Qu Z., Zhang W., Tu L. Hotspots and trends of virtual reality, augmented reality and mixed reality in education field.

[42] Lu J. (2021). Mobile augmented reality technology for design and implementation of library document push system. *Journal of Real-Time Image Processing*.

[43] Cheng K., Furusawa I. The deployment of a mixed reality experience for a small-scale exhibition in the wild.

[44] Bae S., Jung T. H., Moorhouse N., Suh M. (2020). The influence of mixed reality on satisfaction and brand loyalty in cultural heritage attractions: a brand equity perspective. *Sustainability*.

[45] Liu H., Yan Y. An experiential analysis of the influence of critical marketing environment on consumer decision making.

[46] Huang T. L., Liu F. H. (2014). Formation of augmented-reality interactive technology’s persuasive effects from the perspective of experiential value. *Internet Research*.

[47] Haile T. T., Kang M. (2020). Mobile augmented reality in electronic commerce: investigating user perception and purchase intent amongst educated young adults. *Sustainability*.

[48] Morillo P., Orduna J. M., Casas S., Fernandez M. (2019). A comparison study of AR applications versus pseudo-holographic systems as virtual exhibitors for luxury watch retail stores. *Multimedia Systems*.

[49] Adrianto D., Luwinda F. A., Yesmaya V. (2017). Augmented reality implementation in watch catalog as e-marketing based on mobile aplication. *Journal of Physics: Conference Series*.

[50] Poitras E. G., Harley J. M., Liu Y. S. (2019). Achievement emotions with location‐based mobile augmented reality: an examination of discourse processes in simulated guided walking tours. *British Journal of Educational Technology*.

[51] Zhang T., Wang W. Y. C., Cao L. (2019). The role of virtual try-on technology in online purchase decision from consumers’ aspect. *Internet Research*.

[52] Smink A. R., Frowijn S., van Reijmersdal E. A., Neijens P. C. (2019). Try online before you buy: how does shopping with augmented reality affect brand responses and personal data disclosure. *Electronic Commerce Research and Applications*.

[53] Kang J. Y. M. (1989). Augmented reality and motion capture apparel e-shopping values and usage intention. *International Journal of Clothing Science & Technology*.

[54] Li C. Y., Fang Y. H. (2020). I searched, I collected, I experienced: exploring how mobile augmented reality makes the players go. *Journal of Retailing and Consumer Services*.

[55] Huang T. L., Liao S. L. (2017). Creating e-shopping multisensory flow experience through augmented-reality interactive technology. *Internet Research*.

[56] Cruz E., Orts-Escolano S., Gomez-Donoso F., Cazorla M., Mora H., Rangel C. J. (2018). An augmented reality application for improving shopping experience in large retail stores. *Virtual Reality*.

[57] Wakim R. S., Drak A., Miladinovic M., Ozturkcan S. A study of Swedish eyewear retailer’s smartphone-based augmented reality application.

[58] Anderson J. C., Gerbing D. W. (1988). Structural equation modeling in practice: a review and recomm- ended two-step approach. *Psychological Bulletin*.

